# Weighted Average Ensemble Deep Learning Model for Stratification of Brain Tumor in MRI Images

**DOI:** 10.3390/diagnostics13071320

**Published:** 2023-04-02

**Authors:** Vatsala Anand, Sheifali Gupta, Deepali Gupta, Yonis Gulzar, Qin Xin, Sapna Juneja, Asadullah Shah, Asadullah Shaikh

**Affiliations:** 1 Chitkara University Institute of Engineering and Technology, Chitkara University, Rajpura 140401, Punjab, India; 2 Department of Management Information Systems, College of Business Administration, King Faisal University, Al-Ahsa 31982, Saudi Arabia; 3 Faculty of Science and Technology, University of the Faroe Islands, Vestarabryggja 15, FO 100 Torshavn, Faroe Islands, Denmark; 4 Kulliyyah of Information and Communication Technology, International Islamic University Malaysia, Gombak 53100, Selangor, Malaysia; 5 Department of Information Systems, College of Computer Science and Information Systems, Najran University, Najran 55461, Saudi Arabia

**Keywords:** ensembled, weighted average, brain tumor, data augmentation, biomedical, Convolution Neural Network (CNN)

## Abstract

Brain tumor diagnosis at an early stage can improve the chances of successful treatment and better patient outcomes. In the biomedical industry, non-invasive diagnostic procedures, such as magnetic resonance imaging (MRI), can be used to diagnose brain tumors. Deep learning, a type of artificial intelligence, can analyze MRI images in a matter of seconds, reducing the time it takes for diagnosis and potentially improving patient outcomes. Furthermore, an ensemble model can help increase the accuracy of classification by combining the strengths of multiple models and compensating for their individual weaknesses. Therefore, in this research, a weighted average ensemble deep learning model is proposed for the classification of brain tumors. For the weighted ensemble classification model, three different feature spaces are taken from the transfer learning VGG19 model, Convolution Neural Network (CNN) model without augmentation, and CNN model with augmentation. These three feature spaces are ensembled with the best combination of weights, i.e., weight1, weight2, and weight3 by using grid search. The dataset used for simulation is taken from The Cancer Genome Atlas (TCGA), having a lower-grade glioma collection with 3929 MRI images of 110 patients. The ensemble model helps reduce overfitting by combining multiple models that have learned different aspects of the data. The proposed ensemble model outperforms the three individual models for detecting brain tumors in terms of accuracy, precision, and F1-score. Therefore, the proposed model can act as a second opinion tool for radiologists to diagnose the tumor from MRI images of the brain.

## 1. Introduction

A brain tumor, which is regarded as one of the most serious illnesses of the nervous system, is an unexpected and uncontrollable development of brain cells. The Tumor Society estimates that approximately 4 lakh people worldwide are impacted with brain tumors every year [1,2]. Brain tumors can cause a range of complications, including seizures, cognitive problems, and physical disabilities. Early detection and treatment can help reduce the risk of these complications. Detection at an early stage allows for a wider range of treatment options that can help improve the patient’s quality of life, by reducing the need for more invasive treatments and minimizing the impact of the tumor [3,4].

The use of deep learning for the detection of brain tumors is an active field of research that shows significant potential for enhancing the precision and timeliness of brain tumor diagnosis [5,6,7]. Known as a subfield of computer learning, deep learning entails teaching a neural network to spot structures within large datasets. To detect brain tumors, for example, deep learning algorithms can be trained on huge collections of medical photos to recognize the telltale signs of these diseases.

There are several challenges associated with brain tumor detection using deep learning, including the need for large, high-quality datasets, and the difficulty of interpreting the output of the neural network [8]. The availability of enormous medical picture datasets, along with recent advancements in deep learning algorithms, have led to encouraging outcomes in this area. There is hope that future research and development into deep learning-based techniques for brain tumor detection may increase the accuracy and efficiency of brain tumor diagnosis, and ultimately improve patient outcomes.

In deep learning, use of ensemble models can be used to improve the accuracy and robustness of predictions. In the context of brain tumor classification, an ensemble model can help increase the accuracy of classification by combining the strengths of multiple models and compensating for their individual weaknesses. By combining the models, the ensemble model can take advantage of the strengths of each model and mitigate their weaknesses. For example, if a particular model is more prone to overfitting, the ensemble model can compensate by giving less weight to its predictions.

Overall, the use of an ensemble model can improve the accuracy and robustness of brain tumor classification by leveraging the strengths of multiple models and mitigating their individual weaknesses. This can ultimately help clinicians make more accurate and informed decisions about the diagnosis and treatment of brain tumors.

In this research, a weighted average ensemble deep learning model for brain tumor detection is presented. The article’s most significant contributions are as follows:

- A weighted average ensemble model is proposed for the classification of brain tumors by using the grid search for the best combination of weights, i.e., weight1, weight2, and weight3, that are taken for transfer learning model, Convolution Neural Network (CNN) model without augmentation, and CNN model with augmentation, respectively;
- The results of the weighted average ensemble model are compared with the individual model, i.e., transfer learning model, Convolution Neural Network (CNN) model without augmentation, and CNN model with augmentation, in which the proposed ensemble model has outperformed the other individual models;
- Adam optimizer and a 32 batch size were used to evaluate the proposed weighted average ensemble model for brain tumor classification from MRI scans.

The remaining article is prepared as follows: Section 2 demonstrates the literature review, followed by the proposed methodology in Section 3, and the conclusion is shown in Section 4.

## 2. Related Work

The present literature methods are reviewed here. Gill et al. [9] used a VGG19 architecture and achieved an accuracy of 73.0%, precision of 87.0%, sensitivity of 75.0%, and an F1-score of 81.0% on a dataset of 3000 brain MRI images to classify brain tumor. Rajinikanth [10] also used the VGG19 architecture and achieved a higher performance, with an accuracy of 98.17%, precision of 98.50%, sensitivity of 98.75%, and specificity of 97% on a dataset of 1400 MRI images. Khan [11] used both VGG16 and VGG19 architectures and achieved high accuracy on different datasets, with 98.16% on BraTs2015, 97.26% on BraTs2017, and 93.40% on BraTs2018. Khan [12] used a CNN and attained an accuracy of 97.8% on a dataset of 3216 images. Asiri et al. [13] used the VGG19 architecture and attained an accuracy of 98.0% on a dataset of 2870 images. Raj et al. [14] in 2020 used a recurrent neural network technique and achieved an accuracy of 96%, specificity of 98%, and sensitivity of 97%. Poonguzhali et al. [15] in 2019 analyzed 20 patient images using RCNN and SVM classifiers and achieved a sensitivity of 82% and specificity of 99%. Pandian et al. [16] in 2017 analyzed 1000 images using Convnet techniques and attained an accuracy of 97%. Joshi et al. [17] in 2019 used a CNN technique for image analysis and achieved an accuracy of 79.07%. Rao et al. [18] selected patches in each voxel’s plane and trained a CNN. The outputs of each CNN’s final FC layer using softmax were then combined and used to build an RF classifier.

A CNN model is suggested by Kao et al. [19] using the block location data. The ambiguity can be decreased, and the accuracy can be significantly increased by combining the tumor data that has been taken from many advanced networks. To gain more precise anatomical data on brain tumors, Nassar et al. [20,21] fed the CNN model by integrating the image features of long skip-linked lesions. W. Chen et al. [22] showed a separate 3D U-Net model that got around the memory limit by using different 3D convolutions. Wang et al. [23] made a TransBTS structure that worked well with a transformer. Liu et al. [24,25] suggested a customized deep 3D V-Net model based on encoders and decoders that used less memory and computing power and were based on fewer parameters. An attention module with group cross-channel was used to keep track of the most important things [25,26,27]. The suggested work used standard 2017 and 2018 records for research studies. From these two datasets, 2D slices with only the tumorous area were taken.

## 3. Proposed Weighted Average Ensemble Deep Learning Model Architecture 

**Figure 1:**
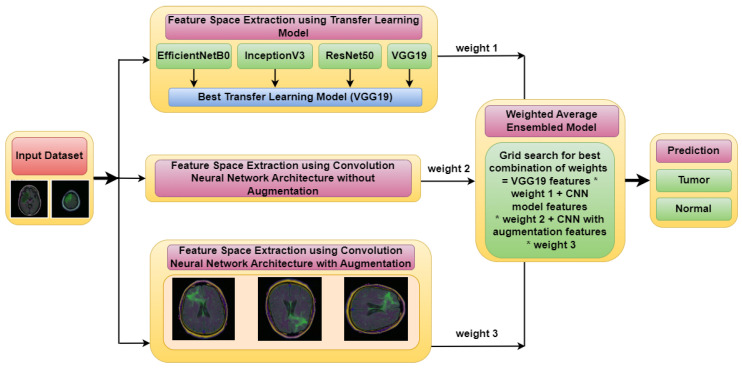
Proposed Weighted Average Ensemble Deep Learning Model for classification of brain tumor.

Figure 1 illustrates the architecture of the proposed Weighted Average Ensemble Deep Learning Model for classifying MRI images of brain tumors. The whole methodology is divided into two phases. The classification is performed using a weighted average ensemble of three models, in which the first model is a transfer learning-based model, the second model is Convolution Neural Network (CNN) model without augmentation and the third model is the CNN model with augmentation. From these three models, three different feature spaces are extracted, which are ensembled, to make an optimized feature space. For this, three different weights, i.e., weight 1, weight 2, and weight 3 are assigned to three different models using a grid search combination to find the best-optimized classification model. By merging the results of several models, an ensemble model can provide more precise forecasts. It is more robust than individual models because if one of the models in the ensemble makes an incorrect prediction, the other models can compensate and provide a correct prediction.

### 3.1. Input Dataset

**Figure 2:**
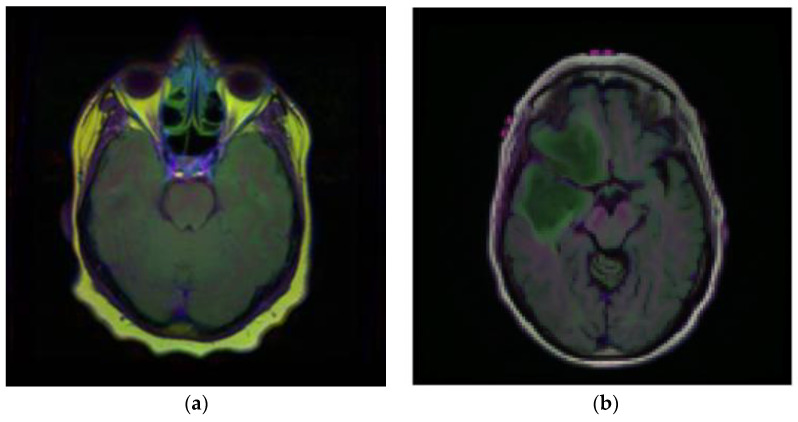
Samples of Brain MRI Images (**a**) Normal Image, (**b**) Tumor Image.

**Figure 3:**
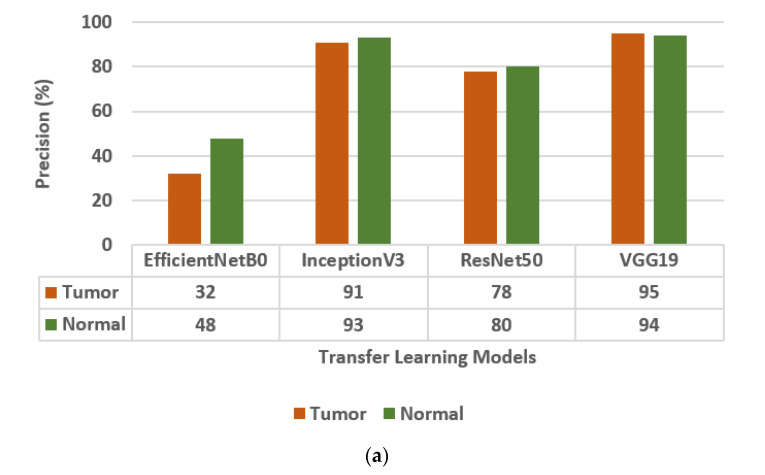

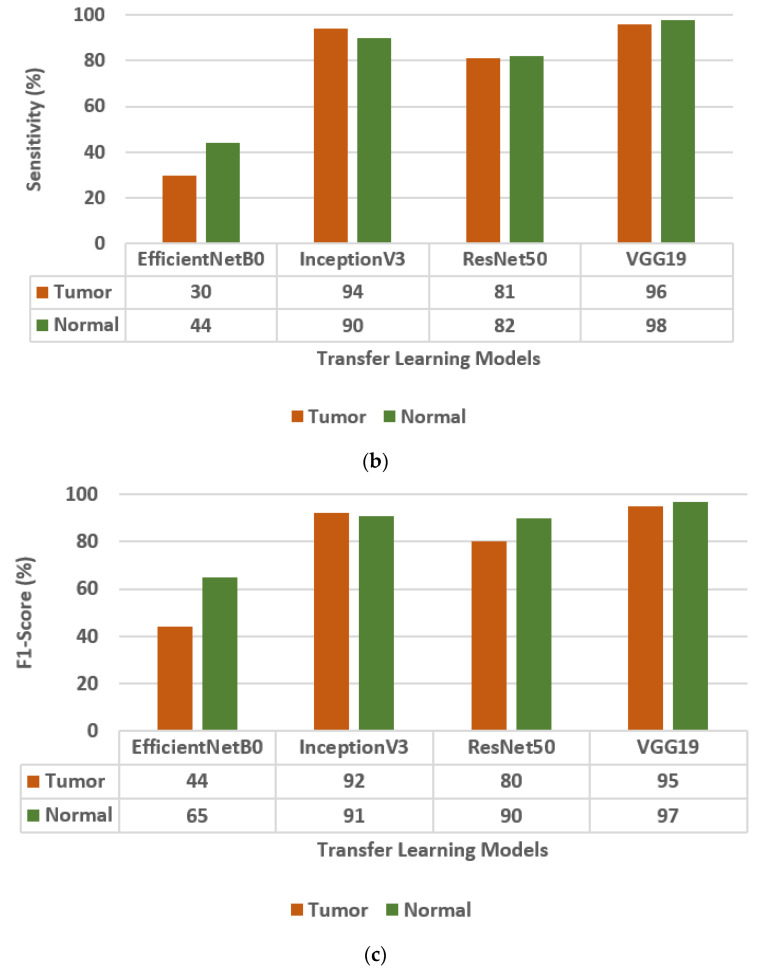
Confusion matrix parameter values of different transfer learning models, i.e., EfficientNetB0, InceptionV3, ResNet50, and VGG19. (**a**) Precision, (**b**) Sensitivity, (**c**) F1-score.

Brain MRI scans from 110 patients with 3929 brain MRI images are included in the dataset using FLAIR abnormalities. Out of the total 3929 dataset images, 90% of the data are used for training and 10% are used for testing. After that, out of the 90% training data, 15% are used for the validation set. Figure 2 illustrates the brain MRI images taken from Kaggle [28,29]. Figure 2a displays the normal image and Figure 2b displays the tumor image of the brain in which two tumor regions are shown with a break in between. It is difficult to segment this break region in the tumor part. The proposed methodology shown in Figure 3 is also segmenting this break part accurately.

### 3.2. Feature Space Extraction Using Three Different Models

In this section, the classification of three models is performed. In model 1, the classification is performed using three different transfer learning models. In model 2, the classification using the Convolution Neural Network (CNN) architecture without augmentation is performed, and in the model 3, the classification is performed using the CNN architecture with augmentation.

#### 3.2.1. Model 1: Classification Using Transfer Learning Models and Evaluation of Best Transfer Learning Model

The different Transfer Learning (TL) models that are used for the classification of brain tumors are EfficientNetB0, InceptionV3, ResNet50 [30], and VGG19. The values of the confusion matrix parameters, such as Precision (PR), Sensitivity (SN), and F1-score (FS) are obtained on all four transfer learning models and are shown in Figure 3. From the analysis of PR, SN, and FS as presented in Figure 3a–c, respectively, it is concluded that the VGG19 model outperforms the other three TL models, i.e., EfficientNetB0, InceptionV3, and ResNet50. The VGG19 model has obtained a precision of 95%, sensitivity of 96%, and F1-score of 95% for brain tumor classes.

#### 3.2.2. Model 2: Classification Using Convolution Neural Network (CNN) Architecture without Augmentation

**Figure 4:**
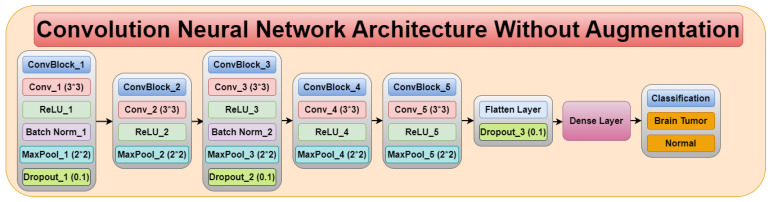
Convolution neural network architecture without augmentation.

The Convolution Neural Network (CNN) architecture consists of five convolution blocks, as shown in Figure 4. Each convolution block consists of different convolution layers, ReLU layer, batch normalization, max pool layer, and dropout layer. Therefore, the CNN architecture consists of five convolution layers, five ReLU layers, two batch normalization, five max pool layers, three dropout layers, flatten layer, and a dense layer. 

**Figure 5:**
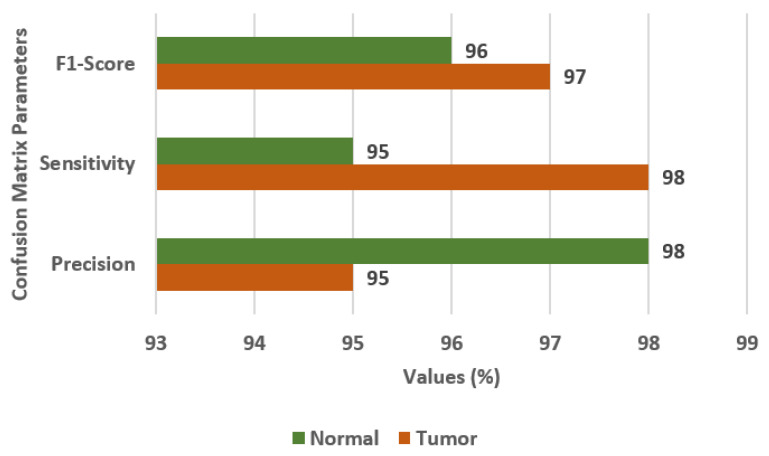
Confusion matrix parameter values for the CNN model.

Figure 5 displays the confusion matrix parameter values obtained using the CNN model. The values of FS, SN, and PR are 97%, 98%, and 95%, respectively, for the tumor class. The CNN model outperformed the VGG19 transfer learning model.

#### 3.2.3. Model 3: Classification Using Convolution Neural Network (CNN) Architecture with Augmentation

**Figure 6:**
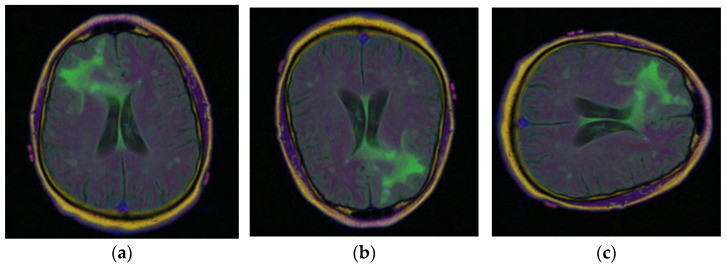
Samples of Brain MRI Images [31]: (**a**) Original Image, (**b**) Vertical Flip, (**c**) Horizontal Flip.

To obtain more, and more varied images of brain tumors, the data augmentation technique is used with the existing images. The different data augmentation techniques [31,32,33] that are applied are vertical flipping and horizontal flipping. Figure 6a displays the original sample of the brain tumor image, Figure 6b displays the vertically flipped image, and Figure 6c displays the horizontally flipped image. 

**Figure 7:**
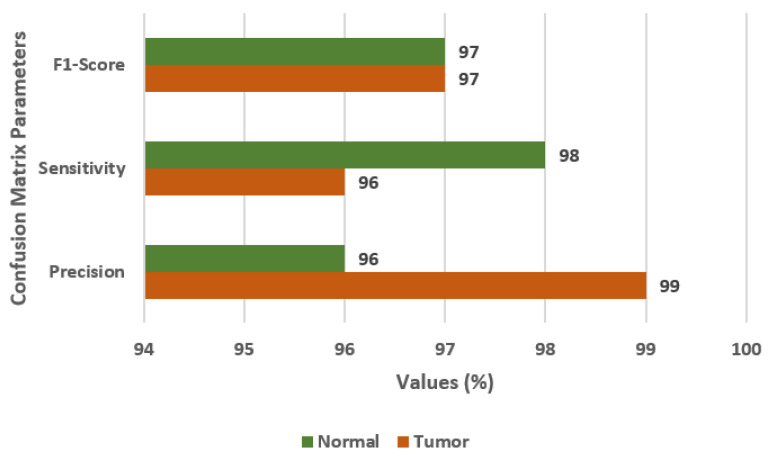
Confusion matrix parameter values for CNN model with data augmentation.

In this section, the results are obtained using the CNN model with augmented images. Figure 7 displays the confusion matrix parameter values on the CNN model with data augmentation. The values of FS, SN, and PR are 97%, 96%, and 99%, respectively. The CNN model with data augmentation, outperformed the previous models.

### 3.3. Classification Using Ensembling of Three Different Models 

The proposed weighted average ensembled model is designed by combining three feature spaces obtained from the TL model, CNN model without augmentation, and CNN model with augmentation. For this, a grid search is performed to find the best combination of weights assigned to three different feature spaces. Weight 1 (wt1) is obtained from the VGG19 TL model, weight 2 (wt2) is taken from the CNN model without augmentation, and weight 3 (wt3) is obtained from the CNN model with data augmentation [34,35,36].

**Figure 8:**
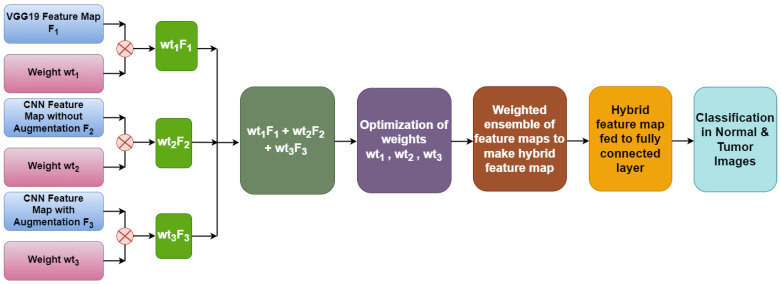
Hybrid feature map generation.

Figure 8 illustrates the weighted ensemble of three feature maps extracted from three different models. These weights are further optimized by using a grid search combination to achieve the maximum accuracy value of the ensemble model. Equation (1) shows the formula of the hybrid feature map for the best combination of weights.

Hybrid feature map = VGG19 feature map F_1_ × wt1 + CNN feature map without augmentation F_2_ × wt2 + CNN feature map with augmentation F_3_ × wt3 (1)

With the help of optimized weights, a hybrid feature map is generated which is further fed to a fully connected layer to determine the classified output.

**Figure 9:**
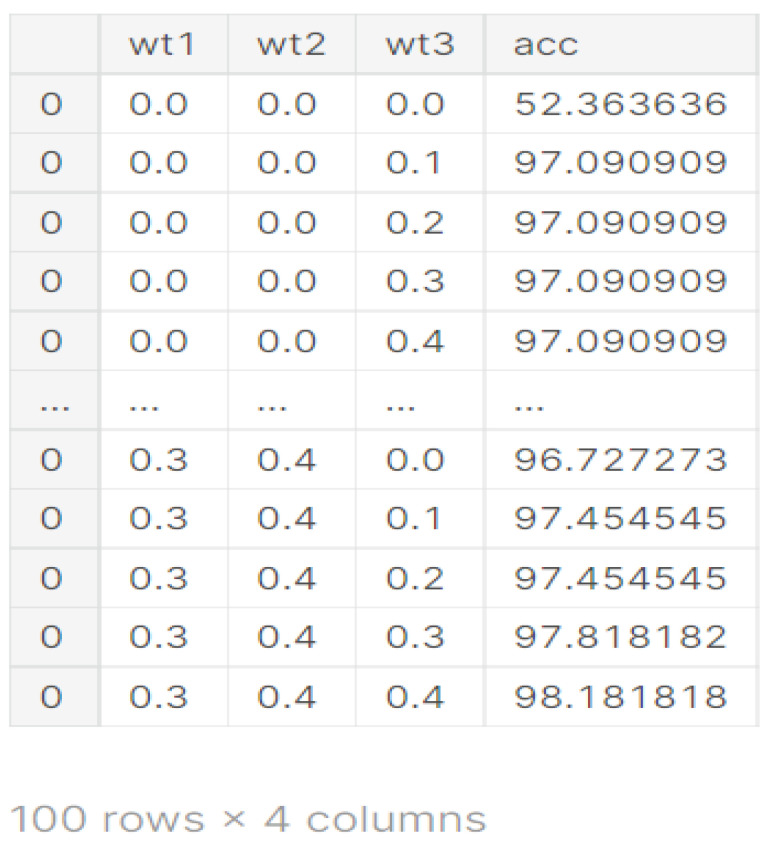
Weights obtained from different models.

From Figure 9, it can be seen that for the different values of wt1, wt2, and wt3, different values of accuracies are obtained. The best value of accuracy 98.18% is obtained on weights 0.3, 0.4, and 0.4 as shown in Figure 9.

**Figure 10:**
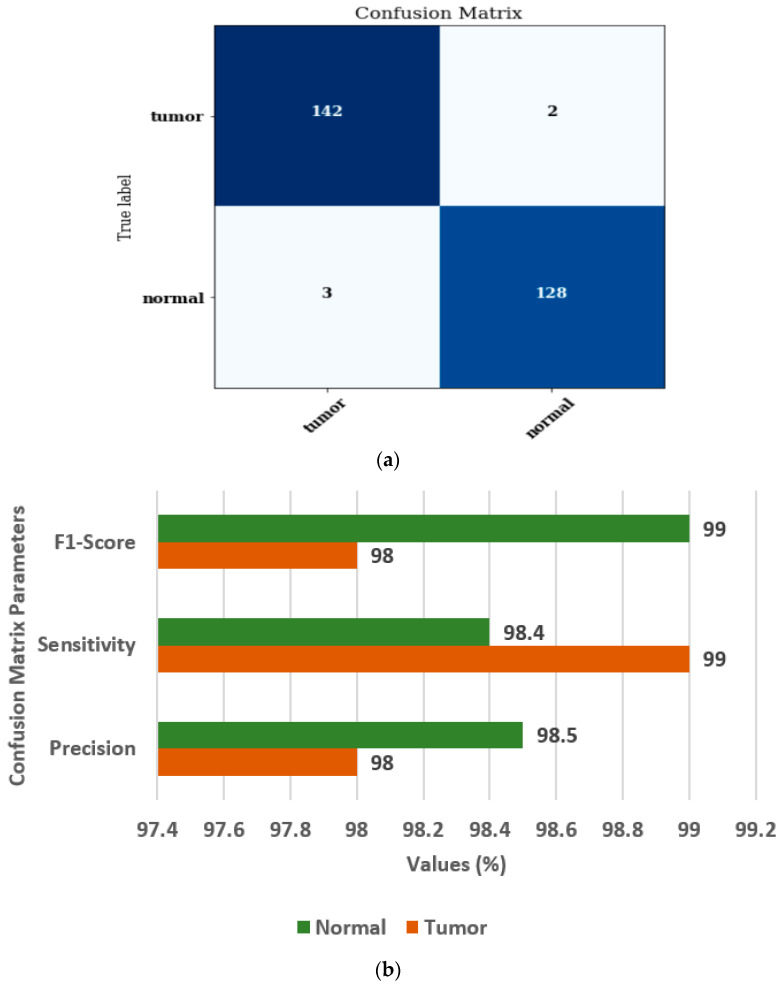
Ensembled model (**a**) Confusion matrix, (**b**) Confusion matrix parameters.

Figure 10 shows the confusion matrix and confusion matrix parameters of the ensemble model [37,38]. Figure 10a displays the confusion matrix for normal and brain tumor classes. Figure 10b displays the values of FS, SN, and PR as 98%, 99%, and 98%, respectively, for the tumor class.

### 3.4. Comparison of Ensembled Model with Individual Models

**Figure 11:**
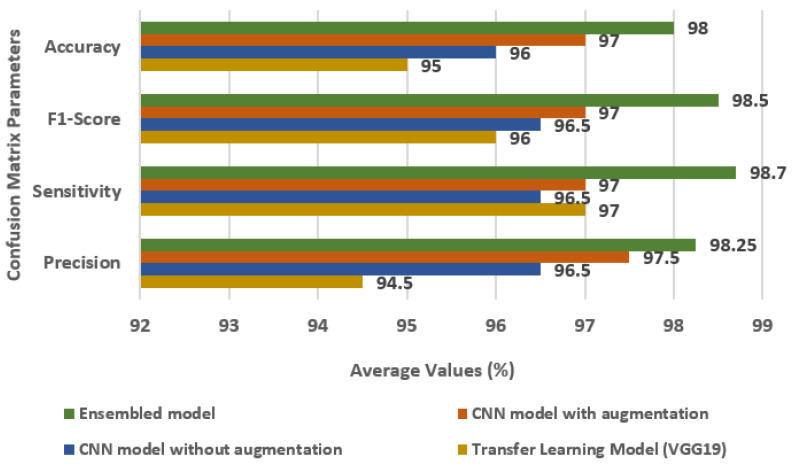
Comparison of Ensembled model with individual models.

Figure 11 displays the comparison of the Ensembled model with individual models, i.e., transfer learning model, CNN model without augmentation, and CNN model with augmentation in terms of accuracy, FS, SN, and PR. For the ensemble model, the values of accuracy, FS, SN, and PR are 98%, 98.5%, 98.7%, and 98.25%, respectively.

### 3.5. Comparison of Ensembled Model with State-of-Art

Table 1 provides a summary of different research studies on medical image analysis, along with the number of images used, the technique employed, and the performance parameters achieved by each study.

Raj et al. [14] used a recurrent neural network and achieved an accuracy of 96%, specificity of 98%, and sensitivity of 97%. Poonguzhali et al. [15] used a RCNN and SVM classifier on 20 patient images and achieved a sensitivity of 82% and specificity of 99%. Pandian et al. [16] used convnet, slicenet, and VGNet on 1000 images and achieved an accuracy of 97%. Joshi et al. [17] used a CNN and achieved an accuracy of 79.07%. Gill et al. [9] used VGG19 on 3000 images and achieved an accuracy of 73.0%, precision of 87.0%, sensitivity of 75.0%, and F1-score of 81.0%. Rajinikanth [10] used VGG19 on 1400 images and achieved an accuracy of 98.17%, precision of 98.50%, sensitivity of 98.75%, and specificity of 97%. Khan [11] used VGG16 and VGG19 on various datasets and achieved accuracies ranging from 93.40% to 98.16%. Khan [12] used a CNN on 3216 images and achieved an accuracy of 97.8%. Asiri et al. [13] used VGG19 on 2870 images and achieved an accuracy of 98.0%.

Finally, the proposed model used a weighted average ensemble model on 3929 images and achieved an accuracy of 98.00%, sensitivity of 98.7%, F1-Score of 98.5%, and precision of 98.25%.

**Table 1:** Review of State-of-the-Art Methods Compared to the Proposed Model.

Ref.	Year	Images	Technique Used	Performance Parameters
Raj et al. [14]	2020	-	Recurrent Neural Network	Accuracy = 96% Specificity = 98% Sensitivity = 97%
Poonguzhali et al. [15]	2019	20 patients	RCNN and SVM classifier	Sensitivity = 82% Specificity = 99%
Pandian et al. [16]	2017	1000	Convnet and slicenet and VGNet	Accuracy = 97%
Joshi et al. [17]	2019	-	CNN	Accuracy = 79.07%
Gill et al. [9]	2022	3000	VGG19	Accuracy = 73.0% Precision = 87.0% Sensitivity = 75.0% F1-score = 81.0%
Rajinikanth [10]	2020	1400	VGG19	Accuracy = 98.17% Precision = 98.50% Sensitivity = 98.75% Specificity = 97%
Khan [11]	2020	-	VGG16 VGG19	Accuracy on BraTs2015 = 98.16% BraTs2017 = 97.26% BraTs2018 = 93.40%
Khan [12]	2022	3216	CNN	Accuracy = 97.8%
Asiri et al. [13]	2022	2870	VGG19	Accuracy = 98.0%
Proposed model	2023	3929	Weighted average ensemble model	Accuracy = 98.00% Sensitivity = 98.7% F1-Score = 98.5% Precision = 98.25%

## 4. Conclusions

Deep learning models can be sensitive to the random initialization of weights, the choice of hyperparameters, and the randomness in the training data. By mixing numerous models trained on distinct portions of the data and using varying hyperparameters, an ensemble model can aid in reducing this unpredictability. In order to classify brain tumors from MRI scans, this research offers a weighted average ensemble deep learning model. The presented work has been estimated on the brain MRI database. It performs classification by using the grid search for the best combination of weights, i.e., weight1, weight2, and weight3 that are taken for the VGG19 TL model, CNN model without augmentation, and CNN model with augmentation, respectively. The proposed ensemble model outperforms the three individual models in relations of accuracy, precision and F1-score, having values of 98%, 98.25%, and 98.5%, respectively. Accordingly, radiologists can use this model as a second opinion resource for making a diagnosis of brain tumors from MRI images.

The study’s inability to generalize findings to other cancer forms attacking MRI pictures is a significant shortcoming. A number of image modalities and segmentation techniques, including the Pyramid Scene Parsing Network (PSPNet), UNet, DeepLab, and Feature Pyramid Network (FPN), can be used in future studies to achieve a good enough approximation of affected brain regions to separate them from healthy ones. It is possible that a combination of modalities, each with its own approach to image registration, will be required to properly display the missing features of image in the patterns over time and execute classification. It is possible that using ensembles would allow for greater precision and accuracy.

## Data Availability

Data Will be available form first author on request.
